# Exploring Newly Qualified Nurses' Perception of Preparedness for Clinical Practice: A Descriptive Qualitative Study

**DOI:** 10.1002/nop2.70328

**Published:** 2025-11-19

**Authors:** Daniel Opotamutale Ashipala, Phellep Ndara Muhora

**Affiliations:** ^1^ Department of General Nursing Science, School of Nursing and Public Health, Faculty of Health Sciences and Veterinary Medicine University of Namibia (UNAM) Rundu Namibia

**Keywords:** clinical practice, fulfilment, perceptions, preparedness, professional goal, professional role

## Abstract

**Aim:**

The aim of this study was to examine the perceived preparedness of newly qualified nurses for clinical practice, with a focus on those factors that contribute to any lack of preparedness. Being prepared for practice is essential for achieving professional goals and reducing unwanted patient and organisational outcomes. The preparedness of newly qualified nurses to practice independently is a topic of debate; however, the factors that cause a lack of preparedness have not been explored thoroughly.

**Design:**

This exploratory study utilised a descriptive and contextual design and thematic analysis.

**Methods:**

The researcher utilised a qualitative methodology for their research. The population of the study was 16 newly qualified nurses, who were chosen through convenience sampling. This study was conducted from August to October 2020 at four campuses that offered a 4‐year Bachelor of Nursing Science (Clinical) (BNSc) Honours Degree. Data were gathered via semi‐structured interviews until the point at which data saturation was reached. Following this, thematic analysis was undertaken.

**Results:**

The analysis of the data led to the development of four themes, that is, student confidence and competence; lack of confidence and competence; resource‐related challenges and suggested changes to improve perceived challenges.

**Conclusion:**

This study's findings have showed that newly qualified nurses experienced some contextual challenges during their four‐year training in fulfilment of their professional roles; they appeared well‐prepared for their clinical roles. Their contextual challenges were as a result of insufficient knowledge, a lack of clinical exposure to some departments, a theory‐practice gap, and insufficient placement time for clinical practice.

**Patient or Public Contribution:**

The findings of this study can be used to develop targeted interventions and ongoing strategies that would inform clinical practice and future curricula development and facilitate improvement of clinical supervision and mentorship.

## 1 Introduction

A clinical nursing education serves as an opportunity for nursing students to learn clinical skills, apply their theoretical knowledge to practice and experience a clinical setting apprenticeship. Ultimately, it exposes the nursing student to the reality of a nursing job's demands and responsibilities. Accordingly, a successful clinical nursing education should provide learning experiences that are constructive and realistic, while ensuring that nursing students are able to practice competently and with confidence (Woo and Li 2020). A nursing student's clinical practice is also aimed at equipping them with the skills to communicate, conduct physical and psychosocial examinations, practice critical thinking, create nursing care plans and attend to other nursing tasks (Nweke et al. 2021).

The Nursing Council of Namibia (NCNA) expects a final‐year honours degree graduate at the point of completion to possess skills such as diagnosing a patient, prescribing medications in the absence of a doctor, and executing interventions to meet the health needs of a patient, or, if needed, referring them to the next level of referral (Government Gazette 2014). The Gazette also postulated that a well‐prepared final‐year student should be able to ensure the maintenance of a patient's fluids, manage their electrolyte and acid–base balance, and supervise and maintain their oxygen supply. As midwifery is included in this degree, a student should also be able to provide antenatal and labour care, as well as puerperium interventions and an episiotomy if required.

These clinical skills are acquired by nursing students through their clinical placements, which is why they are compulsory. Clinical placements also expose students to real human patients while simultaneously having to interact with community members (Adjei et al. 2018). During clinical placement periods, nursing students exercise different practical skills under the supervision of professional nurses (Tuomikoski et al. 2020). According to the Nursing Council of Namibia, newly graduated nurses are expected to be responsible and accountable for their actions while practising nursing care (HPCNA 2014). The responsibility of the School of Nursing at the University of Namibia (UNAM), however, is to train nurses in the competencies they will require after graduating, as well as prepare them to adapt to an ever‐evolving healthcare system.

Preparedness in practice indicates effectiveness in performing tasks that are appropriate to a given situation and profession. Preparedness is essential for achieving professional goals and reducing unwarranted practice outcomes. A well‐prepared student nurse is able to practice effectively in a clinical setting (Rosa and Maniago 2018); thus, nursing graduates are expected to employ their knowledge and skills in the clinical environment as soon as they enter into practice (Haruzivishe and Macherera 2021). Practice preparedness is therefore essential for every nursing student, but particularly for those who choose to practice in clinical nursing (Rosa and Maniago 2018). This is because the on‐the‐job performance of newly graduated nurses can be affected by a lack of confidence, which can jeopardise patient care and safety (Rusch et al. 2019).

The responsibility for newly qualified nurses' preparedness for clinical practice is complex, in part because it is shared between the academic and clinical healthcare settings (Leufer and Cleary‐Holdforth 2020). The first year of independent practice sees new nurses typically focusing on applying the knowledge and skills they acquired in school. This has been described as “stressful” and “overwhelming” given their desire to deliver quality care (Hussein et al. 2017). The preparedness of newly qualified nurses to independently practice has recently been debated (Salem 2021), as university nursing programmes have raised concerns about the inadequate clinical skills of their graduates (Mekgoe et al. 2019; Xu et al. 2018). In addition, concerns have been raised by the graduates of such programmes themselves (Donough and Van Der 2018), as well as the healthcare providers with whom they find employment following graduation (Kaihlanen et al. 2019). It is therefore vital to determine nursing graduates' preparedness to fulfil their duty of care, given the obstacles and unforeseen events that can arise in a healthcare system. This study, therefore, sought to explore and describe the lived experiences of newly graduated nurses from our local university on their readiness to assume their professional roles as newly qualified nurses. The study was deemed important as it would inform clinical practice and future curricula development and facilitate improvement of clinical supervision and mentorship.

## 2 Study Aim

### 2.1 Aim

The study's aim was to explore and describe the perceived preparedness of newly qualified nurses from our local university for clinical practice as qualified nurses.

### 2.2 Design

The research is a cross‐sectional, qualitative, descriptive study. The data were analysed inductively and were collected via in‐depth, face‐to‐face interviews. Therefore, a descriptive, exploratory, and qualitative design was applied as it enabled the newly qualified nurses to relay their experiences regarding their perceived preparedness of final‐year nursing students from our local university for clinical practice as qualified nurses. The use of a qualitative research methodology enabled the researchers to gain an in‐depth understanding of the perceived preparedness of newly qualified nurses from our local university for clinical practice as qualified nurses (Levitt et al. 2018). This was done through semi‐structured interviews, where the participants shared their own unique, individual descriptions and interpretations on the basis of their experiences from a descriptive perspective (Creswell 2013; Polit and Beck 2017).

### 2.3 Context

This study was conducted from August to October 2020 at four campuses that offered a 4‐year Bachelor of Nursing Science (Clinical) (BNSc) Honours Degree. A total of 450 nursing students were registered for their final year across the four campuses in Namibia. The degree is only offered full time, with students alternating 2 weeks learning theory in class and 2 weeks in a clinical setting for practical sessions (Shatimwene et al. 2020). The curriculum is approved by three accreditation bodies, that is, the National Council of Higher Education (NCHE), the Nursing Council of Namibia (NCN) and the Namibia Qualification Authority (NQA). The competence‐based curriculum is based on the constructivist theory, that is, it is founded on the belief that “people can construct knowledge and transform it for meaningful use and integration into life roles.” The lecturers for this programme must hold a relevant master's degree or a doctorate. The clinical instructors and preceptors teach and assess students on their practical competency, while socialising them to the profession.

### 2.4 Sample

The sample size for qualitative studies can typically be determined by data saturation, which occurs when no new information is obtained and redundancy is observed (Polit and Beck 2017). In this study, data saturation was achieved with 16 participants, who were selected through convenience sampling. The second author (FA) liaised with a nurse educator from each of the four campuses to convey study information to all newly qualified nurses, without determining who should participate in the study. Each of the participants received a participant information sheet and was given the opportunity to ask questions, decide voluntarily if they wanted to participate in the study, and sign a consent form. The participants were free to withdraw at any time to ensure conscious and ethical involvement. The interviewer had no prior contact with the newly qualified nurses. In other words, the interviewer did not know the participating newly qualified nurses. All the nursing students who took part in the study broadly displayed the demographics of the wider population. This relationship did not have any influence on the research findings as there was no power imbalance between the researcher and interviewees. Not all interested participants who met the inclusion criteria and were willing to participate in the study were interviewed, as data saturation was reached with the 16th participant. The inclusion criteria for this study were (1) being a newly qualified nurse for a 4‐year Bachelor of Nursing Science (Clinical) (BNSc) Honours Degree and (2) agreeing to participate in the study by signing a consent form. A written invitation letter/flyer that explains the purpose of the study was shared through email with the university management to distribute to potential participants through various media such as emails and to be placed on the notice boards of the university, campus tea rooms and board rooms, in order for information about the study and recruitment of the potential participants, and if willing, to contact the researcher. Potential participants who responded to the researcher's invitation were contacted via email or by telephone for appointments. A record was kept of interested persons' contact details and the researchers invited them to an information session. Persons who met the inclusion criteria were invited to participate, and informed consent was requested should they agree to participate. Although the researcher is a novice, he was trained in qualitative research interviewing techniques and conducted a pilot interview with newly qualified nurses from the same sampling unit to ensure that the interview process was of high quality and yielded meaningful data. During the actual qualitative data collection, the supervisor for the researcher was available to assist the researcher as needed.

### 2.5 Data Collection

Following approval by the University Research Ethics Committee, convenience sampling was employed to select 16 students who were in their final year and were willing to sign a written consent to participate. An interview guide was developed on the basis of the literature review, the central research question, and the research objectives. A pilot interview was conducted with three participants from the same sampling population to test the interview guide's ability to generate relevant data. Given the interview guide's suitability, no revisions were made to the interview guide, and data from the pilot interview did not form part of the study. The face‐to‐face interviews were conducted individually, with data saturation being reached on the 16th interview, that is, the interviews were no longer yielding new information (Grove et al. 2013). The interviews, which were semi‐structured and ranged between 30 and 40 min each, were audio‐recorded and transcribed verbatim. The interview guide consisted of open‐ended questions, which enabled the researcher to examine the newly qualified nurses' perceptions regarding their readiness for practice. The data collected were kept in a computer encrypted with a PIN, which only the researcher and their supervisor had access to. Open‐ended questions were used to allow the participants to share their experiences in detail (Moser and Korstjens 2018). The interviews, which lasted for 40–50 min, were conducted in English at a venue that was convenient for the participants. The audio recordings, interview transcripts, and field notes were stored in a password‐protected and encrypted computer, which will be deleted after 2 years if the study is published. If it is not published, the data will be deleted after 5 years. The interview guide asked the following questions:

1. Based on your own opinion, how are the theoretical and clinical competencies being addressed by the Bachelor of Nursing Science?
2. What is your opinion on competence regarding your preparedness to start practising?
3. Have you encountered any challenges in acquiring clinical competence?
4. What changes would you suggest regarding preparing graduates for clinical practice?
5. Is there anything else you would like to add?

Possible probing questions

Can you elaborate on that?

What are your thoughts regarding that?

What more can you share on that?

### 2.6 Data Analysis

The analysis of data in this study followed the six steps described by Braun and Clarke (2019), namely, “(1) Familiarise yourself with your data; (2) Assign preliminary codes to your data to describe the content; (3) Search for patterns or themes in your codes across the different interviews; (4) Review themes; (5) Define and name themes; and (6) Produce your report” (Braun and Clarke 2019, 60). By describing and presenting a compelling account of the data‐driven findings, both across and within themes, the author addressed the research questions and provided relevant information beyond a straightforward description of the themes. After the initial coding, the researcher met with the co‐coder (data analyst) to discuss the findings from each of their perspectives. There was general agreement on key issues surrounding the codes, sub‐themes, and themes, yet the final wording of the findings did not exactly match the initial findings or the findings from the co‐coder. Despite this, the key aspects were retained and the findings matched most of the sub‐themes from the co‐coder. Any differences can be attributed to the researcher's condensing of the themes and further aligning them to the data. An in‐depth recording of all evidence was also kept.

### 2.7 Trustworthiness

Trustworthiness in this qualitative study was ensured by adopting (Lincoln and Guba 1985), which assesses credibility, dependability, confirmability and transferability. In this research, credibility was ensured through lengthy engagement and member checking. The study made use of peer debriefing, and the data collected were recorded via audio tapes and notes. The researcher describes the data collected as precisely as possible, and thick descriptions of the participants' experiences are made available. The verbatim transcripts were kept for an audit trail to ensure confirmability, with comparisons being made between the transcripts in order to check for similarities and ensure credibility. Additionally, both the dependability and the confirmability of the research were ensured through the use of an experienced independent coder with a doctorate in nursing education, in which qualitative research was used. Each analyst conducted their own thematic analysis, and any discrepancies or potential biases were discussed in detail. Decisions were reached through consensus to ensure a thorough and unbiased interpretation of the data. This multi‐layered approach helped to enhance the validity and credibility of the data analysis process. As the semi‐structured interviews consisted of open‐ended questions, they ensured transferability (Guba 1981). To ensure dependability, the data collected in this study were recorded; transcripts can be made available upon enquiry. The data gathered were also compared with relevant literature on the phenomenon under study. Additionally, the study includes a dense, thick description of the methodology steps taken, thereby ensuring dependability.

### 2.8 Ethical Consideration

The university's Ethical Research Review Committee approved this study (reference number: SoNREC 22/2019). Consent was gathered from the interviewees, who had the freedom to remove themselves from the study should they so wish, without any repercussions. Each participant was assigned a code to guarantee their anonymity, and all were given equitable and fair treatment. The data that were gathered have stayed confidential; only the researcher, their supervisor and the independent coder have access to them. The research participants had the right to withdraw from the study at any time, and their participation was voluntary. Assurance was given that the data would be kept confidential and used only for the purposes of this research. There were no risks or direct benefits related to participation in this study.

## 3 Results

### 3.1 Participants' Demographic Information

All of the participants were newly qualified nurses working at the selected teaching hospital (see Table 1). Each of them had completed a 4‐year Bachelor of Nursing Science (Honours) (Clinical) degree at the School of Nursing and Public Health in Namibia. Five of the participants were male and 11 were female. This gender variance is typical of nursing schools. All the participants were under the age of 33.

**TABLE 1:** Demographic profile of the participants.

Participant	Age	Gender	Marital status
Participant 1	33	Male	Single
Participant 2	24	Male	Single
Participant 3	21	Male	Single
Participant 4	24	Male	Single
Participant 5	33	Male	Single
Participant 6	24	Female	Single
Participant 7	28	Female	Single
Participant 8	30	Female	Married
Participant 9	23	Female	Single
Participant 10	22	Female	Single
Participant 11	25	Female	Single
Participant 12	23	Female	Single
Participant 13	22	Female	Single
Participant 14	22	Female	Single
Participant 15	22	Female	Single
Participant 16	32	Female	Single

### 3.2 Presentation and Discussion of Findings

Four categories emerged as the main perceived preparedness of final‐year nursing students for clinical practice: (1) Student confidence and competence; (2) Lack of confidence and competence; (3) Resource‐related challenges; and (4) Suggested changes to improve perceived challenges (see Table 2). Each of these categories is presented and discussed below.

**TABLE 2:** Emerging themes and themes sub‐themes.

Themes	Sub‐themes
3.1. Theme 1: Student confidence and competence	3.1.1. Allocated simulation period acquiring skills and knowledge 3.1.2. 2 weeks of theory taught for practical aspects 3.1.3. 2 weeks of clinical practice in hospital wards or clinics
3.2. Theme 2: Lack of confidence and competence	3.2.1. Competency in using mechanical ventilators 3.2.2. Competency in suturing wounds 3.2.3. Competency in radiotherapy and chemotherapy 3.2.4. Ability to carry out blood transfusions and invasive procedures 3.2.5. Competency in management, administration, and leadership
3.3. Theme 3: Resource‐related challenges	3.3.1. Clinical practice by qualified staff differs from theory 3.3.2. The library has insufficient books for nursing students 3.3.3. First aid training is not fully addressed in the curriculum 3.3.4. Clinical placement practical hours are insufficient for knowledge and skills transfer
3.4. Theme 4: Suggested changes to address the perceived challenges	3.4.1. Exposure of students to all departments 3.4.2. Practical time be extended to a month for mastering clinical skills 3.4.3 Clinical preceptors or instructors to do follow‐ups at clinical settings 3.4.4 University to organise internships 3.4.5. First aid to be added during next review cycle

#### 3.2.1 Theme 1: Student Confidence and Competence

This theme relates to the participants' reflections on how the theoretical and clinical competencies are being taught by the Bachelor of Nursing Science (BNSc). The sub‐themes of this theme include: Allocated simulation period for acquiring skills and knowledge; 2 weeks' theory taught for practical aspects; 2 weeks clinical practice in hospital wards or clinics.

##### 3.2.1.1 Allocated Simulation Period for Acquiring Skills and Knowledge

Students expressed that the practical competencies of the BNSc are addressed through the allocation of students to simulation rooms.

Then through simulation, mostly simulation is the method they use to teach. (P 1)

The Bachelor of Nursing Science allocates simulation period during theory time for student nurses to acquire clinical skills and knowledge. (P 10)

##### 3.2.1.2 Two Weeks Theory Taught for Practical Aspects

Participants revealed that theoretical applications are addressed during theory blocks, which last 2 weeks each.

We are allocated for two weeks on theory, where the theoretical aspect of nursing is taught. (P 2)

So, at campus we're given lectures to tackle the theoretical part, information is provided, and we are prepared on how to carry procedures when we go on practical and it is a two‐week block. (P 6)

##### 3.2.1.3 Two Weeks Clinical Practise in Hospital Wards or Clinics

Participants revealed that clinical applications are addressed through the allocation of clinical blocks, which take place in hospital wards and clinics.

We are placed on practicals for two weeks either in the hospital wards or clinics. (P 2)

We are allocated for two weeks at a clinical setting, where we do our practice under the supervision of RNs. (P 3)

#### 3.2.2 Theme 2: Lack of Confidence and Competence

Under this theme, the participants expressed their perception of their preparedness to start practising. Sub‐themes under this theme are as follows: Competency in using mechanical ventilators, Competency in suturing wounds, Competency in radiotherapy and chemotherapy, Ability to carry out blood transfusions and invasive procedures and Competency in management, administration and leadership.

##### 3.2.2.1 Competency in Using Mechanical Ventilators

A participant revealed a lack of competency when it comes to operating mechanical ventilators.

I am still not competent enough to operate a ventilator machine. (P 3)

During my four years of training I only managed to nurse one patient on a ventilator, so operating a ventilator on my own will be so difficult due to a lack of exposure. (P 12)

I had a couple of quite difficult times during the first months working with a ventilator. (P 1)

##### 3.2.2.2 Competency in Suturing Wounds

Another participant felt that there is an element of incompetence in suturing wounds.

Honestly, I am somehow not prepared to start practicing because I am not competent enough to suture wounds. (P 4)

I am only competent in suturing minor wounds but if it's coming to deep wound suturing I only sutured three which I am still struggling to do on my own. I still need some supervision when doing it. (P 7)

##### 3.2.2.3 Competency in Radiotherapy and Chemotherapy

A lack of preparedness was evident among the participants in terms of practising radiotherapy and chemotherapy.

I am not well prepared to start practicing because I am not competent in caring for patients on radiotherapy and chemotherapy. (P 5)

I am not prepared enough because I am not knowledgeable about chemotherapy and radiotherapy care for patients, I don't know how to do it since I was not exposed. (P15)

##### 3.2.2.4 Ability to Carry out Blood Transfusions and Invasive Procedures

In this sub‐theme, the participants had mixed reactions about their preparedness to administer blood transfusions and perform invasive procedures. Some participants highlighted that they were not prepared for practice in this regard.

I'm not entirely prepared, because I'm not used to working alone without supervision.

More especially when it comes to the blood transfusion procedure. (P 9)

I am not prepared enough because I am not competent in blood withdrawing in paediatrics and also in inserting an IV cannula. (P 7)

However, some participants expressed that they were prepared to start practising in performing invasive procedures.

I am competent in cannulating and withdrawing blood and I'm prepared to start working as a registered nurse. (P 1)

I am prepared to start practicing because I am competent in cannulation and withdrawing blood. (P 8)

##### 3.2.2.5 Competency in Management, Administration and Leadership

Some participants revealed that they are competent in management, administration, and leadership.

I can do well with administration, be it finance or whichever way which involves leadership. I am competent. (P 8)

I am prepared to start practicing; I am competent in management and administration and gained competence in this aspect. (P 16)

I am well prepared in management but I still feel that I will encounter a challenge in prescribing medications, more especially if I end up working at a healthcare centres or clinics where are no doctors but RNs alone. (P 9)

#### 3.2.3 Theme 3: Resource‐Related Challenges

This theme reflects what participants in this study expressed as their challenges with regard to the Bachelor of Nursing Science degree. The sub‐themes that emerged included clinical practice by qualified staff differs from theory; books in the library insufficient for large numbers; first aid component not fully addressed in the curriculum; and practical block insufficient for knowledge and skills transfer.

##### 3.2.3.1 Clinical Practice by Qualified Staff Differs From Theory

Some participants expressed concern over the gap between theory and clinical practice in the clinical blocks. It was stated that the knowledge acquired during lectures at the university is not implemented in the same way in the clinical areas, which can lead to confusion among students and result in incompetence.

Some of the content or information of procedures we are being taught in theory are implemented differently at the hospital setting. (P 3)

Some of what we are told in class during theory differs at clinical practice, for example babies presenting with fever; at school we are taught to prioritise such babies but at the hospital we are being told as long as the patient has no seizures at the present time, that baby should remain in the queue even if for a long period before attended. (P 6)

##### 3.2.3.2 The Library Has Insufficient Books for Nursing Students

Some participants expressed concern regarding the lack of books in the university's library, arguing that there are not enough to cater to the large number of nursing students.

The other challenge is the library [there] is lack of enough books so that's a huge challenge …, you know we need books. (P 2)

Books in the library are not enough for the large number of students, making it difficult for students to read more about diseases, conditions and procedures. (P 1)

##### 3.2.3.3 First Aid Training Not Fully Addressed in the Curriculum

This sub‐theme revealed that first aid is not being fully addressed in the BNSc curriculum, leading to students not having the necessary skills to handle emergencies.

We are studying nursing, but we do not do first aid; at least if we can do first aid, it will help us understand the management of emergency cases better. (P 13)

If we could have done first aid as a module, we could have enough experience and skills that we can use to manage serious cases; now we have to learn new things on our own. (P 2)

When something happens sometimes we don't know what to do; we are not prepared for it, because it was not done in our training. (P 3)

##### 3.2.3.4 Clinical Placement Practical Hours Insufficient for Knowledge and Skills Transfer

This sub‐theme is a description of the participants' views regarding what they consider to be inadequate time spent on the practical blocks.

The practical block of two weeks duration was not enough for me to grasp all the clinical knowledge and skills. (P 3)

It is very difficult to learn hands‐on skills in the clinical setting because of the short clinical placement. I didn't complete my procedures; I had a very limited time. (P10)

The time we spent in the clinical area is very short. (P 6)

Two clinical blocks are not enough for us to acquire all the necessary skill that we need; I suggest clinical blocks be increased to two or three weeks per allocation. (P 16)

#### 3.2.4 Theme 4: Suggested Changes to Improve Preparedness

This theme reflects the participants' suggestions on how to improve students' preparedness to start practising after completing their degree. Sub‐themes that emerged included: Exposure of students to all departments; practical time be extended to a month for mastering clinical skills; clinical preceptors or instructors to do follow‐ups in clinical settings; the university to organise internships; and first aid to be added during the next review cycle.

#### 3.2.5 Exposure of Students to All Departments

Participants suggested that nursing students should be exposed to all the departments in a hospital.

I think students should be exposed to all departments at the hospital setting, so that they are familiar with the procedures that are done in those specific departments. (P3)

I personally suggest that it will be important if all students are allocated to all hospital departments just to familiarise themselves with different ward routines and procedures. (P 6)

##### 3.2.5.1 Practical Time to Be Extended to a Month for Mastering Clinical Skills

Participants suggested that the practical blocks be extended to a month in order for students to gain adequate practical competence and to be prepared.

Also, the practical time could be extended to at least a month, so that students can master the clinical skills very well. (P 3)

I feel the practical block should be made even one month, which is four weeks in a specific department. With the four weeks, I think it's better you might learn more. (P11)

##### 3.2.5.2 Clinical Preceptors or Instructors to Do Follow‐Ups in Clinical Settings

Participants suggested that clinical instructors conduct follow‐ups with students in clinical areas in order to provide adequate supervision to students.

I think the clinical instructors or preceptors can strictly follow‐up on the fourth‐year nursing students when they are at clinical areas, for supervision and to make sure they are prepared for work the following year. (P 1)

The clinical instructors are not coming to the hospital or wards often to follow up on their students. They have to come often and see how the students are doing and how they are behaving in the wards, and also to understand in which areas the students need help most. (P12)

##### 3.2.5.3 University to Organise Internships

Participants suggested that the university could organise internships for final‐year nursing students once they have passed the programme but are still waiting for graduation.

I feel with nursing, there should be an internship even for three months; if we are three months in the practical setting, we will learn and be exposed to a lot of things. (P 11)

I think the Nursing Council should help organise internships for the new graduates; it would make it easier for us to gain practical experience and be more prepared to go in the field. (P 15)

##### 3.2.5.4 First Aid to Be Added During the Next Review Cycle

Participants suggested that the first aid course or module be included in the Bachelor of Nursing Science curriculum to improve the preparedness of final‐year nursing students.

First aid could be added to the nursing programme for students to be competent. (P13)

I really think First Aid should be part of the new curriculum update. It is something we use in almost every setting and we should be confident in it from the start of our career. (P 3)

First Aid training will help us with the confidence to assist patients properly during emergencies. (P 10)

## 4 Discussion

### 4.1 Student Confidence and Competence

Some participants reported that students are allocated theory and simulation periods; hence, their theoretical and practical competencies are nurtured. Uslu et al. (2020) concluded that the utilisation of simulation rooms by nursing students gives them confidence, which is applied in their clinical practice. Participants in this study reported that they have 2‐week theoretical blocks, after which they apply their knowledge during full‐time 2‐week practical blocks, which is as per the findings of qualitative studies by Drateru (2019) and Shatimwene et al. (2020).

### 4.2 Lack of Confidence and Competence

The findings of this study revealed that the participants did not have enough clinical skill exposure in certain areas, including the use of mechanical ventilators, suturing wounds, radiotherapy and chemotherapy, and were thus unprepared for those aspects of clinical practice. Although participants in this study felt that they lacked competence in skills, including operating mechanical ventilators, “practising” chemotherapy and radiotherapy and suturing, these skills are typically associated with the role and scope of advanced nursing practice or experienced nurses. Surprisingly, these participants expressed awareness of the need for them to possess these skills, which employers would not expect them to have at the point of employment. This is incongruent with existing literature around preparedness for practice of similar cohorts, which cite more fundamental core nursing responsibilities that newly qualified nurses, approaching the terminal phase of their programme, typically grapple with.

Accordingly, the Nursing Council of Namibia does it does not require newly qualified nurses in this particular jurisdiction to have a degree of capability and competence in these skills. Therefore, the above perceived lack of preparedness is in alignment with a study conducted by López‐Entrambasaguas et al. (2019), which found that students rated using mechanical ventilation as the area they were least competent in. Guner's (2015) study reported similar findings, whereas Son et al. (2016) reported that students were not competent in suturing wounds. López‐Entrambasaguas et al. (2019) reported that students in their study rated themselves poorly on administering chemotherapy medications as well as carrying out blood transfusions.

Encan and Akin (2019), meanwhile, reported that over 80% of novice nurses need in‐service training on blood transfusions and administration because they lack competence in these areas. In the same vein, Shamshirian et al. (2017) reported that the majority of new nurses require in‐service training on blood transfusions. In addition, Usher et al. (2015) discovered that many final‐year students are afraid, anxious, and uncomfortable when asked to do invasive procedures. This situation is indicative of a serious lack of preparedness among independent registered nurse practitioners.

Despite this, the majority of the participants indicated that they felt competent enough in management, administration, and leadership tasks. Simmons' (2011) findings also revealed that nursing students were competent and prepared with regard to these three skills, namely, unit administration, management, and leadership skills, and were prepared to start working as qualified nurses. It is generally accepted that all novices will feel nervous carrying out procedures for the first time or accept a greater level of responsibility for areas such as medicine administration, so there is a certain level of appropriateness in this anxiety. Besides the Simmons' (2011) findings, a few participants indicated that they were not ready to start practising as qualified nurses because they felt incompetent in the administration of medication, especially if they worked at healthcare centres or clinics where there are no doctors. The findings of a study conducted by López‐Entrambasaguas et al. (2019) emphasised the importance of making pharmacology a year module instead of being taught for one semester.

### 4.3 Resource‐Related Challenges

The findings of this study revealed that participants encountered challenges related to a theory‐practice gap, detailing scenarios where qualified registered or staff nurses implemented procedures in clinic areas differently from how students were taught them by lecturers during their theory blocks. A study by Drateru (2019) showed similar results, whereby students raised concerns that staff nurses did not always carry out procedures in the correct manner or in the way they were taught in theory. Furthermore, some participants in this study reported that there were too few books in the library for the number of students, thus making it difficult for them to learn about relevant conditions and procedures.

Matshotyana et al. (2015) reported that this is a common challenge for many universities, especially in Africa. Additionally, some interviewees in this study stated that a first aid component was not included in the BNSc programme, leaving students ill‐equipped to perform first aid. Similar challenges were reported by (Registerednursern.com, 2015). A concern was also raised by some participants that the current practical block's duration is not enough to acquire sufficient knowledge and skills. This is also as per Helgesen et al. (2016), who reported that students do not spend sufficient time in clinical areas, undermining their competence and confidence.

### 4.4 Suggested Changes to Improve Preparedness

Participants in this study suggested that students need to be exposed to all departments in a hospital to familiarise themselves with all the procedures that are done in each department. This is in line with Motsaanaka et al. (2020) and Makhene and Ally (2020), who suggested that it should be compulsory for student nurses to be allocated to all clinical departments during their training, as this would expose them to learning opportunities that would enable them to acquire clinical skills and become competent and safe professional nurses. It was also suggested by the interviewees in this study that the practical blocks be extended to a month in order for them to master clinical skills. Muhora and Ashipala (2021) also recommended in their study that the existing 2‐week block system be extended to 4 weeks to provide enough time for students to apply their learned theory into practice in a clinical setting. It was further suggested that clinical preceptors or instructors conduct follow‐ups in a clinical setting.

This is in line with Phuma‐Ngaiyaye et al. (2017), who noted that more clinical knowledge is gained when students are supported by their preceptors in clinical settings, resulting in more confidence and competence. One participant suggested that the university should organise internships for final‐year nursing students once they have passed the programme but are awaiting graduation. According to Nguyen (2024), internships help students to learn about and understand their patients better, as they learn to recognise their needs and respond accordingly. A first aid course or module should also be included in the BNSc curriculum, which corresponds with the findings of Muhora and Ashipala (2021), who recommended that universities should add a first aid module as a core module in the first year of a nursing degree, as this would prepare students better for an emergency situation.

## 5 Limitations and Areas for Further Research

The study findings were limited by the number of participants interviewed, thus limiting the generalisability of the results. Nevertheless, as this was a descriptive study that provides initial exploratory data, it was not our intention to generalise. Additionally, the study was based on the student nurses from one local university, thus the study findings cannot be generalised to student nurses studying at other universities as the context may differ. The use of convenience sampling may have introduced bias, as the participants may not have fully represented all nurses working in the region. Additionally, the study's reliance on self‐reporting through interviews may have led to social desirability bias, with the participants providing responses they perceived to be socially acceptable. Triangulation of data from multiple sources or methods could have enhanced the credibility and validity of the findings.

The study could have benefitted from incorporating perspectives from nurse educators, industry players and other stakeholders involved in regulatory bodies for nursing education and training in the country. Newly qualified nurses could focus their research on various aspects of their transition and professional development. Key areas should include the impact of simulation‐based education, the effectiveness of preceptorship programmes, the challenges of rural placements and the experiences of nurses in critical care. By focusing on these areas, newly qualified nurses can contribute to a better understanding of the challenges and opportunities associated with their transition to professional practice and improve the overall support and retention of nurses within the healthcare system.

### 5.1 Recommendations

On the basis of the study findings, the following practical recommendations for curriculum development and clinical training are made:

- For effective curriculum development and clinical training, prioritise student needs, establish clear learning outcomes, design engaging learning experiences and implement robust assessment strategies.
- Incorporate real‐world scenarios, utilise diverse teaching methods and provide regular feedback to enhance student learning.
- Involve stakeholders, align with national and international standards and continuously evaluate and improve the curriculum.
- Utilise high‐fidelity simulations to provide safe and controlled environments for practising clinical skills.
- Pair students with experienced clinicians for mentorship and guidance.
- Incorporate community‐based learning experiences to expose students to diverse patient populations and healthcare settings.

## 6 Conclusion

The findings of the current study showed that most of the participants at UNAM were not prepared to start practising as registered nurses, yet the majority described a general lack of expertise, which was impeding their preparedness. Participants felt they lacked competence in skills including operating mechanical ventilators, chemotherapy, radiotherapy and suturing; however, these skills are typically associated with more experienced nurses. It should be noted that their lack of perceived preparedness for clinical practice was based on skills including operating mechanical ventilators, chemotherapy, radiotherapy and suturing; however, these skills are typically associated with more experienced nurses. Therefore, their lack of preparedness should be interpreted under these typical skills that are not expected from newly qualified nurses but those with more experience.

A greater focus on ongoing education for graduates may assist on the basis of an understanding that not all graduates emerge with advanced skills. Hence, there is a need to further embed in the education of the nurses some realistic expectations and plans for ongoing education. Additionally, lack of competence in specific nursing procedures is a result of insufficient knowledge, not enough clinical exposure, a theory‐practice gap, and insufficient time in clinical practice. The findings from this study can be used to develop targeted strategies and interventions that can be used by nursing schools to strengthen their programmes.

## Author Contributions

Daniel Opotamutale Ashipala drafted the article, made critical revisions on the paper and supervised the study. Phellep Ndara Muhora conceived, designed, collected and analysed the study. All authors have agreed on the final version and meet at least one of the following criteria (recommended by the ICMJE).

## Conflicts of Interest

The authors declare no conflicts of interest.

## Data Availability

Data in this study can be made available from the corresponding author at reasonable request.
